# Resident and Non-resident Father Involvement, Coparenting, and the Development of Children’s Self-Regulation Among Families Facing Economic Hardship

**DOI:** 10.3389/fpsyg.2022.785376

**Published:** 2022-02-21

**Authors:** Lauren E. Altenburger

**Affiliations:** Department of Human Development and Family Studies, The Pennsylvania State University, Sharon, PA, United States

**Keywords:** father involvement, parenting, self-regulation, coparenting, non-resident fathers

## Abstract

Self-regulation, or the ability to effectively manage emotions and behavior, is a critical skill to develop in early childhood. Children living in a context of economic hardship are at an increased risk for developing self-regulation difficulties. However, few studies have comprehensively examined how multiple aspects of the caregiving environment, including fathers’ parenting and coparenting quality, may contribute to child self-regulation. Thus, this study applied a family systems perspective to examine whether coparenting and resident and non-resident fathers’ reports of parenting quantity and quality were associated with observations of children’s self-regulation. Participants were drawn from the Embedded Developmental Study (*n* = 257) of the Three-City Study, a longitudinal study of children and families facing economic hardship. At Wave 1, when children were 2–4 years old, reports of parenting (i.e., quantity and quality) and coparenting (i.e., support) were obtained. At Wave 2, when children were 3–6 years old, children participated in a snack delay and gift wrap task, which assessed their self-regulation. Multi-group path analyses indicated that resident fathers’ harsh parenting at Wave 1 predicted decreased levels of self-regulation at Wave 2. Non-resident fathers’ reported hours of involvement at Wave 1 predicted greater levels of self-regulation at Wave 2. Additionally, supportive coparenting among families with a non-resident father predicted greater self-regulation. Supportive coparenting was not associated with child self-regulation in families with a resident father. The implications for research focused on facilitating positive father–child relationships in diverse family contexts are discussed.

## Introduction

Of the more than 12 million children under 3 years of age living in the United States, 24% live in families with a household income below the federal poverty line. An additional 22% of children live in families with a household income between 100 and 200% of the poverty line (Aber, 2012). Although growing up in a low socioeconomic environment is associated with several risks, some children overcome the challenges and exhibit adaptive developmental outcomes. A risk and resilience framework suggests that children who thrive may possess personal or environmental resources that promote their success (Jenson and Fraser, 2016). One personal resource is children’s self-regulation, or the ability to effectively manage and coordinate behaviors, thoughts, and emotions in the pursuit of a goal (Carver and Scheier, 2016). Children with high levels of self-regulation can appropriately and flexibly adjust their actions to the demands of the situation, which is advantageous in meeting expectations across a variety of formal (i.e., school) and informal (i.e., home) settings (Eisenberg et al., 2010; Drake et al., 2014). In low socioeconomic contexts, children who develop adaptive self-regulation exhibit greater resilience and school readiness (Raver, 2012). Thus, understanding factors that promote positive self-regulation in this context is beneficial for promoting positive developmental outcomes in young children.

Notably, the quality of the caregiving environment can support adaptive self-regulation, especially during the early years of life. Emerging research examining the contributions of mothers’ parenting to child self-regulation in low socioeconomic contexts has revealed more positive parenting supports better self-regulation (i.e., Brophy-Herb et al., 2012; Julian et al., 2019). However, surprisingly few studies have considered how coparenting, or the extent to which parents support or undermine each other’s parenting strategies, and fathers’ parenting may positively contribute to the development of self-regulation. This study applied a family systems perspective (Cox and Paley, 2003) to consider how multiple family relationships may be associated with child self-regulation. In particular, fathers’ parenting quantity (i.e., hours of involvement), fathers’ parenting quality (i.e., self-reports of authoritative or harsh parenting behaviors), and coparenting relationship quality (i.e., support) were examined as predictors of young children’s self-regulation in a sample of families facing economic hardship. Notably, statistical models included mothers’ parenting quality as a control variable to more stringently evaluate whether fathers’ parenting and coparenting contributed to child self-regulation beyond the contributions of mothers’ parenting. Additionally, this study examined whether the links between fathers’ parenting, coparenting, and child self-regulation varied between families with a resident father and families with a non-resident father.

## The Development of Self-Regulation

Self-regulation is a general term that refers to the variety of strategies that a child draws upon to achieve a goal. Early childhood is an important period for developing self-regulation, as children who develop strong self-regulation skills are often better equipped to achieve long-term goals (Zelazo and Carlson, 2012). The behavioral aspect of self-regulation includes controlling impulses, monitoring behavior, and inhibiting a dominant response (Calkins, 2007) and emerges during the first few years of life (Diamond et al., 2002; Holmboe et al., 2008). Children are required to draw upon their early behavioral self-regulation strategies in many daily activities, such as when they refrain from eating a forbidden treat or raise their hand rather than shouting out the answer during class (Gagne and Saudino, 2016). Thus, self-regulation strategies are particularly beneficial in early childcare settings. For example, children with greater levels of behavioral self-regulation are better able to maintain concentration, persevere, and ultimately achieve a goal (Macdonald et al., 2014). Children who struggle to establish self-regulation by middle childhood, in contrast, often experience other social and learning difficulties later in life (Ciairano et al., 2007; Wåhlstedt et al., 2008). Thus, identifying family relationships that best support children’s self-regulation is vital in promoting children’s long-term positive adjustment and success.

### The Role of Parenting

Notably, the neural networks underlying self-regulated behavior are remarkably plastic and can be shaped by environmental experiences during the early childhood years (Gunnar and Fisher, 2006). As such, high-quality parenting, characterized by warmth, sensitivity, and responsiveness, is theorized to facilitate the development of positive self-regulation skills (Rochette and Bernier, 2014). In contrast, low-quality parenting can induce stress and overwhelm the young child’s emerging self-regulation system (Blair, 2010). Indeed, researchers have reported a positive association between the quality of mothers’ parenting behaviors and better self-regulation skills in young children (Bernier et al., 2010; Choe et al., 2013). However, considerably less is known about the consequences of fathers’ parenting quality and quantity of involvement for children’s self-regulation. Modern United States fathers are more involved in their children’s lives than ever before (Schoppe-Sullivan and Fagan, 2020). Thus, considering the contribution of both mothers’ and fathers’ parenting may yield important insights into the underpinnings of child self-regulation.

One of the most widely used conceptualizations of father involvement is the Lamb, Pleck, Charnov, and Levine tripartite model (Lamb et al., 1985, 1987), which introduced engagement, accessibility, and responsibility as key components of father involvement. As referenced in Pleck’s (2010) updated version of the model, fathers’ positive engagement activities may be particularly important in supporting child adjustment. Theorists propose fathers support child adjustment, including self-regulation skills, by facilitating the child’s “exploration system” and encouraging children to interact with their environment and take risks (Grossmann et al., 2002; Paquette, 2004). Additionally, when fathers engage in highly stimulating positive engagement (i.e., rough and tumble play), they challenge children’s emerging regulatory system, which, in turn, supports self-regulation (StGeorge and Freeman, 2017). Notably, intensive, highly stimulating father–child play is not universal. For example, in foraging societies, father–child playful interactions are rarely observed, and fathers more often engage in “intimate caregiving” (Hewlett et al., 1998).

Consistent with the view that Western fathers may engage in parenting behaviors that foster child self-regulation, a handful of studies have examined associations between aspects of fathers’ parenting and child self-regulation. Collectively, these studies have indicated that more positive parenting, including physical play (Bocknek et al., 2017), child-oriented play (Owen et al., 2013), and self-reports of parenting quality (Roskam et al., 2014; Lucassen et al., 2015) are positively associated with child self-regulation. However, studies that have included fathers tend to draw form higher-socioeconomic status samples with resident fathers (Bernier et al., 2012; Meece and Robinson, 2014), which includes children at the lowest risk for developing self-regulation difficulties. Non-resident and low-income fathers are often overlooked in child development studies due to the challenge of recruiting and tracking them longitudinally (Tamis-Lemonda and McFadden, 2010).

However, incorporating non-resident fathers is vital for obtaining a complete picture of how both parents contribute to child self-regulation across diverse contexts. The number of children with a non-resident father in the United States continues to grow. Data from the United States Census indicate that 31% of children under 18 years do not live with both of their biological parents (Census, 2016). Notably, the proportion of children living with a non-resident father varies dramatically by socioeconomic status. Among families with an annual household income below $50,000, 41.6% of children live with their mother only and have a non-resident father. For families with an income between $50,000 and $74,999, 21.1% of children live with their mother only and have a non-resident father. At even higher income levels (i.e., family income $100,000 or higher), 5.7% of children live with their mother only and have a non-resident father (Census, 2020). Often with more limited resources (i.e., time, money), low-income non-resident fathers face unique barriers to their involvement in childrearing and, as a result, report lower levels of involvement compared to resident fathers. This trend has been reported in Western countries (i.e., the United States; Mincy et al., 2015) and in other cultural contexts, where higher percentages of children have a non-resident father (i.e., the Caribbean; Gray and Brown, 2015).

Notwithstanding the barriers to involvement, social expectations for non-resident fathers to take an active role in their child’s life are strong, and non-resident fathers are involved in their children’s lives at increasing rates (Carlson and McLanahan, 2010; Mincy et al., 2015). Further, accumulating evidence indicates high quality non-resident father involvement contributes to improved child well-being (Carlson, 2006; Adamsons and Johnson, 2013; Nepomnyaschy et al., 2014). For example, data from the Early Head Start Research and Evaluation study of low-income fathers revealed that children with a stable relationship with a non-resident father scored higher on cognitive functioning measures than children without a stable relationship (Vogel et al., 2006). Additionally, non-resident father involvement is positively associated with better academic outcomes for children (Miller et al., 2020). However, the consequences of specific aspects of non-resident fathers’ parenting for children’s self-regulation remain unclear (Roy and Smith, 2013). Therefore, including both resident and non-resident fathers in studies of self-regulation would support a comprehensive view of the role of fathers in the development of self-regulation.

### The Role of Coparenting Relationship Quality

Family systems theorists view the family as a complex network of interdependent relationships (i.e., mother–child, father–child, sibling) that, when considered together, create a whole greater than the sum of its various parts (Cox and Paley, 2003). Thus, beyond the parenting-child relationship, relationships at the family level are a key context in which children’s self-regulation develops. The coparenting relationship, or the extent to which the child’s caregivers can effectively work together and coordinate child-related responsibilities (Feinberg, 2003), is considered the “executive subsystem” of the family (Minuchin, 1974). The quality of the coparenting relationship can vary between families—with some parents exhibiting warm, supportive coparenting relationships and other parents undermining each other and competing for their child’s attention. As such, the quality of interparental interactions can theoretically “spillover” and influence the quality of other family subsystems.

A growing body of research has revealed direct associations between coparenting relationship quality and child outcomes (Teubert and Pinquart, 2010). In particular, when parents do not support each others’ parenting strategies, child adjustment suffers (Nandy et al., 2021). In contrast, when coparenting support is high, children exhibit fewer internalizing and externalizing difficulties (Farr et al., 2019). So what explains the direct link between coparenting and child outcomes? The emotional security hypothesis suggests that when parents support each other’s parenting strategies and the overall atmosphere between parents is calm and respectful, children have a greater sense of security (Davies et al., 2002). Conversely, when parents undermine and disrespect each other’s parenting, children experience increased feelings of stress and reactivity that may interfere with their ability to self-regulate (Kuhlman et al., 2018).

Research examining associations between coparenting and child self-regulation remains largely understudied. However, in one notable exception, researchers reported a link between supportive coparenting and children’s self-regulation among families living in Portugal (Baptista et al., 2018). In particular, lower levels of cooperation predicted more self-regulation difficulties in children. Although this work is an important first step in advancing the field’s understanding of how the coparenting relationship is associated with child self-regulation, the study design was cross-sectional. Additionally, it did not include mothers’ parenting quality as a predictor. The current study builds upon this research by examining longitudinal associations between mothers’ and fathers’ parenting, coparenting relationship quality, and child self-regulation in a sample of United States families experiencing economic hardship. In addition, in the current study, children’s earlier levels of self-regulation are included as a control variable. This approach enables a more thorough examination of whether early parenting and coparenting contribute to an *increase* or *decrease* in children’s self-regulation over time.

## The Present Study

Grounded in family systems theory, the central goal of this study was to more comprehensively consider how multiple aspects of the caregiving environment are associated with children’s self-regulation. In particular, fathers’ parenting (i.e., quality and quantity) at Wave 1 was examined as a predictor of subsequent child self-regulation at Wave 2. It was expected that children would exhibit greater self-regulation when fathers are more involved and take an authoritative approach in their parenting. Fathers’ harsh discipline strategies and low levels of involvement, in contrast, were expected to be associated with decreased self-regulation. Beyond fathers’ parenting behaviors, the family system is an important context in which children’s self-regulation develops. Although largely unexplored, coparenting relationship quality (i.e., supportive coparenting) was also considered as a predictor of child self-regulation. It was expected that greater coparenting relationship quality, characterized by support between parents, would be associated with greater self-regulation in children.

I also examined whether there were differences between children with a resident father and children with a non-resident father. Because non-resident fathers do not live with their children, they may face unique barriers to being involved in their children’s lives. Prior research has revealed positive contributions of non-resident father involvement for child outcomes (Adamsons and Johnson, 2013). However, few studies have examined children’s self-regulation as an outcome. In this study, I expected that non-resident father involvement would be positively associated with child self-regulation. However, the extent to which quantity versus quality of involvement might be associated with child self-regulation was an exploratory question. Additionally, an exploratory question was whether the effects of coparenting relationship quality would be the same for children with a resident versus a non-resident father. Supportive coparenting may be beneficial for children’s self-regulation regardless of their father’s residential status. Alternatively, supportive coparenting might be more beneficial for some children than others. For example, supportive coparenting might be even more valuable in circumstances where fathers are non-resident. When fathers are not physically residing with their children, supportive coparenting might be especially important in creating a positive emotional climate in the home. Low levels of supportive coparenting, in contrast, might be even more negatively associated with child self-regulation when fathers are non-resident.

Certain child characteristics were included in the final model to better disentangle the consequences of fathers’ parenting for child self-regulation. Namely, child gender was included as a control variable, as prior research has indicated that female children outperform males on measures of self-regulation (Matthews et al., 2009). Additionally, child age was controlled, as older children are likely better able to self-regulate (Raffaelli et al., 2005).

## Materials and Methods

### Participants and Procedures

Participants were drawn from Welfare, Children, and Families: A Three-City Study, a longitudinal and multi-method study of the well-being of low-income children, families, and communities in Boston, Chicago, and San Antonio during the post-welfare reform era (Angel et al., 2012). Approximately 2,400 children living in low-income families (ages 0 to 4 or 10 to 14 years old), defined as a household income less than 200% of the federal poverty threshold, were obtained using stratified, random sampling techniques.

Although the child and the child’s primary female caregiver (typically the biological mother) were the focus of the larger study, efforts were taken to provide additional depth to evaluations of child adjustment. In particular, the Three-City Study also included an Embedded Developmental Study (EDS) component, which focused on children aged 2 to 4 years at Wave 1 and children aged 3 to 6 years at Wave 2 of the longitudinal study, as this is a sensitive developmental period in which patterns of behavior and ways of responding to the environment are established. This developmental period also requires parents to learn effective strategies of responding to their child and providing appropriate warmth, discipline, and opportunities for learning (Winston et al., 1999). The EDS was developed to gather a more detailed understanding of various environments and processes that influence child adjustment during the early childhood period (Winston et al., 1999). Principal investigators of the Three-City Study designed the EDS to provide detailed information about father involvement and childcare.

To supplement the principle points of data collection in the Three-City Study, the primary method of measurement in the EDS was observational assessments, in addition to a detailed interview with the child’s biological father and mother (at Wave 1 only). As an incentive to participate in the EDS, each participant (i.e., mother, child care provider, and father) received $30. In addition, the child received a small toy for participating in the videotaped assessments.

All children ages two to four and their parents were invited to participate in the EDS. Of the approximately 2,400 children who were included in Wave 1, approximately 31% were between 2 and 4 years of age (*n* = 737). Of eligible children included in the EDS (*n* = 737), 626 mothers completed the required EDS measures (R.R. = 84.9%). When EDS-eligible mothers provided contact information and researchers were also able to locate fathers, interviews were conducted with the focal child’s biological father. Of eligible fathers who were reached and agreed to participate in the study (*n* = 272), eight fathers reported that they had not had any contact with the focal child in more than 12 months. These fathers were not asked questions about the quality of involvement with their children and, therefore, were not included in the present study (Little et al., 2014)^1^. After accounting for missing data on variables of interest, 257 fathers were included in the final sample. Of participating fathers, 106 were resident (41.25%) and 151 were non-resident (58.75%) at Wave 1, when parenting and coparenting were assessed. Of families with a non-resident father (*n* = 151), 9.9% of children had a stepfather or maternal boyfriend father figure. Three maternal boyfriends lived in the household.

From Wave 1 to Wave 2, 26 fathers changed from residential to non-residential. Nineteen fathers changed from non-residential at Wave 1 to residential at Wave 2. There were no significant differences in Wave 2 self-regulation scores among children who experienced a change in their father’s residential status compared to the rest of the sample in the snack delay task [*t*(189) = −0.63, *p* = 0.53] or the gift wrap task [*t*(189) = −0.69, *p* = 0.49].

Additionally, among the full sample (*n* = 257), 192 families had data on child self-regulation at Wave 2 (74.7%). Attrition analyses indicated that there were not statistically significant differences in Wave 1 child self-regulation in the snack delay task [*t*(216) = −0.38, *p* = 0.71], Wave 1 child self-regulation in the gift wrap task [*t*(212) = 0.30, *p* = 0.77], child age [*t*(255) = 0.12, *p* = 0.91], or fathers’ education level [*t*(255) = 1.62, *p* = 0.11] between families with Wave 1 and Wave 2 data and families with Wave 1 data only.

Graduate students and upper-level undergraduate students with training in child psychology or education were hired for coding children’s self-regulation at Waves 1 and 2, as principal investigators believed they would be more aware of the constructs of interest. The team of coders trained to assess child-self regulation included seven coders. Of the seven coders, four coders were fluent in Spanish. Each coder participated in 10 weeks of training, during which coders were introduced to the larger study and discussed issues related to family process, child development, and cultural sensitivity. After coders learned the entire coding scheme, 10 tapes were coded on all variables and interrater reliability was established. After training, approximately 25% of cases were double-coded, and the trainer checked scores and coders met to discuss discrepancies and come to an agreement. Note, all data in the current study, including the observational codes of self-regulation, were obtained from the EDS. The study author only had access to publicly available, de-identified data. The data are publicly available: https://www.icpsr.umich.edu/web/DSDR/studies/4701.

### Comparing Participating and Non-participating Fathers

Prior to conducting the main analyses, preliminary analyses were undertaken to examine potential similarities and differences between participating (*n* = 257) and eligible, non-participating fathers (*n* = 480). In cases where fathers did not participate, a majority were non-resident fathers (92.29%), as indicated by mother reports. Of fathers who did not participate and were non-resident, 37.16% lived outside the city and 9.3% were in jail or in an institution other than jail. Additionally, independent samples *t*-tests were conducted to clarify the ways in which participating fathers might or might not have been similar to non-participating fathers. Although data were not available for non-participating fathers’ education level, there was not a statistically significant difference in maternal education between the two groups [*t*(721) = −0.46, *p* = 0.6424]. There was not a statistically significant difference in household income between the two groups [*t*(590) = 0.43, *p* = 0.67]. Finally, for all fathers, mothers responded to the question “About how often has [FATHER] seen [CHILD] during the past 12 months?” on a scale of 1 (never in the past 12 months) to 5 (almost every day). On the whole, participating fathers saw their child more frequently (*M* = 4.09, *SD* = 1.07) than non-participating fathers (*M* = 3.12, *SD* = 1.36) [*t*(431) = −7.58, *p* < 0.001].

### Measures

#### Wave 1

##### Parenting

Both mothers and fathers reported on their relationship with the focal child and their parenting practices. Parents reported the degree to which they agreed with various types of parenting strategies (1 = *definitely true* to 4 = *definitely false*). Seven items assessed authoritative parenting practices (i.e., “I give [CHILD] a chance to explain [his/her] side before punishing [him/her]” or “I try to show that I understand [CHILD]’s feelings when I punish [him/her] for misbehaving” or “I try to make rules which take [CHILD]’s individual needs into consideration”). Two items assessed harsh parenting practices (i.e., “I think that a good spanking is sometimes needed to make [CHILD] understand” or “I spank [CHILD] when [he/she] has done something really wrong”). Items were recoded so that higher responses indicated a higher endorsement of the items. Then, authoritative and harsh parenting items were averaged separately to create authoritative and harsh parenting composite variables for each parent. The reliability for each scale are provided: fathers’ authoritative (α = 0.56) and harsh (α = 0.78) parenting behaviors and mothers’ authoritative (α = 0.67) and harsh (α = 0.81) parenting behaviors.

##### Fathers’ Quantity of Involvement

Fathers were asked to estimate how many hours they were currently taking care of the focal child per week by responding to the question, “These days, do you ever take care of your child?” and “About how many hours?” A standardized composite variable was computed to indicate fathers’ current level of involvement in childcare.

##### Coparenting Support

Mothers and fathers reported on coparenting support via two items that ranged from 1 (*none*) to 4 (*a lot*). Fathers responded to the question, “These days, how much does your involvement make things easier for [CHILD]’s mother or make her a better parent?” In contrast, mothers responded to the question, “How much does father involvement make things easier for you or make you a better parent?” Additionally, fathers responded to the question, “These days, how much does your financial or material support, such as money, housing, or things like diapers or clothes for [CHILD], help [his/her] mother?” Mothers responded to the question, “How much did father financial and material support such as money, housing, or things like diapers for [CHILD] help you?” Cronbach’s alpha indicated acceptable internal consistency for mothers’ (α = 0.88) and fathers’ reports (α = 0.81). Fathers’ perceptions of coparenting support and mothers’ perceptions of coparenting support were averaged (*r* = 0.54, *p* < 0.001).

##### Self-Regulation

Self-regulation was assessed using two delay of gratification tasks designed to measure children’s ability to inhibit a dominant response in order to achieve a specific goal. The structure and coding of both self-regulation tasks were based on the Gift Wrap and Snack Delay tasks developed by Kochanska et al. (1996). In the Gift Wrap task, the experimenter tells the child that he or she will receive a present. However, the experimenter wants to wrap it, and the child is instructed not to peek while the experimenter noisily wrapped the present for 50 s. Children’s specific peeking behaviors were coded on a scale of 0 (child gets out of his/her chair and goes over to field investigator) to 7 (child does not try to peek). Additionally, time lapsed to first peek and time lapsed to turning around, defined as when the child shifts hips to look, were coded. A composite self-regulation score in the gift wrap task was computed by standardizing and taking the mean of children’s behavior, time at first peak, and time to turn around in the gift wrap task, with higher scores indicating better self-regulation. Cronbach’s alpha indicated acceptable internal consistency (α = 0.93).

Children’s self-regulation was also assessed using the Snack Delay task. In this task, children were asked to wait until they heard a bell to retrieve an M&M candy. Four trials (varying in length from 20, 40, 60, and 30 s) assessed two components: (1) the time from the start of trial (when the M&M was given to the child) until the research assistant lifted the bell, signifying the end of the procedure is near, and (2) the time from when the research assistant lifted the bell to the time when the research assistant rang the bell, signifying the end of the procedure. For each trial, coders entered two scores: (1) the difference between the start and end time, and (2) the specific behaviors exhibited by the child in the task. The end time was documented when either the bell was rung or the child ate the M&M, as noted as when the M&M passed the child’s lips—whichever event came first. The child’s uninhibited behaviors were coded on a scale of 0 (*child eats M&M during Part I*) to 10 (*child waits until bell rings to eat M&M*). If multiple behaviors occurred, the most uninhibited behavior was coded. A single composite variable indicating children’s overall behavioral regulation in the snack delay task was created by standardizing and taking the mean of children’s behavior regulation and composite proportion of time waited to eat the snack. Cronbach’s alpha indicated acceptable internal consistency (α = 0.77).

##### Resident Status

Mothers reported fathers’ residential status via a single question: “Does father live in the same household as child?” (1 = *yes*; 2 = *no*).

#### Wave 2

##### Self-Regulation

Two similar delay-of-gratification tasks were used to assess children’s ability to inhibit a dominant response. In the Gift Task, the child was given a can of Play-Doh and instructed not to touch it. The research assistant explained that he or she would look for a second can of Play-Doh to give the child. Coders assessed how long children waited to touch the can of Play-Doh, how long children waited to open the can of Play-Doh, and how well the child refrained from touching the can of Play-Doh on a scale of 1 (*child takes Play-Doh out of can*) to 10 (*child does not touch the can*). In instances where the child exhibited multiple behaviors, the least controlled behavior was coded. Only the first 50 s of the task was coded. A composite score indicating children’s overall behavioral regulation in the Gift Task was calculated by standardizing the behavior code, the time reflecting how long the child waited to open the gift, and the time reflecting how long the child waited to touch the gift, and then taking their average. Cronbach’s alpha indicated acceptable internal consistency (α = 0.84).

Children were also asked to wait until a bell rang to retrieve an M&M candy. Six trials that varied in length were administered. Each trial included two parts: (1) The time from the start of the trial until the research assistant lifted the bell, signifying the end of the procedure is near, and (2) the time from when the research assistant lifted the bell to the time the research assistant rang the bell, signifying the end of the procedure. Each trial included a score to indicate the difference between the actual start and end time (the time at which the bell was rung or the M&M candy was eaten – whichever came first), and a score to indicate specific behaviors that occurred during each trial. The M&M candy was considered “eaten” at the moment the candy passed the child’s lips—even if the child still had his/her fingers on it or later took it out of his/her mouth. During various timed trials, the timer began the moment the M&M was placed in front of the child by the research assistant. Behaviors were coded from 0 (*child eats M&M during Part I*) to 10 (*child waits until bell rings to eat M&M*). If the child exhibited multiple behaviors, then the lowest number (most uninhibited behavior displayed) was coded. A composite variable indicating children’s overall behavior regulation in the snack delay task was created by standardizing the mean behavior code and the mean proportion time and taking their average. Cronbach’s alpha indicated acceptable internal consistency (α = 0.63).

##### Control Variables

Child age, child gender, and father education level were included as control variables.

### Analytic Plan

First, descriptive statistics, including the mean, standard deviation, and range, were calculated for variables of interest. Differences in children’s self-regulation at Wave 2 by fathers’ residential status were evaluated using independent samples *t*-tests.

Second, path analyses were performed using Mplus version 8.4 statistical modeling software (Muthén and Muthén, 2017). Model parameters were estimated with Full Information Maximum Likelihood estimation (FIML) with standard errors that are robust to non-normality (MLR estimator) to examine whether fathers’ parenting quality (i.e., authoritative and harsh parenting) and quantity of involvement and supportive coparenting at Wave 1 were associated with children’s self-regulation at Wave 2, while controlling for children’s earlier levels of self-regulation and mothers’ parenting quality (i.e., authoritative and harsh parenting). Child gender, age, and fathers’ education level were also included as control variables. The errors between child age and child self-regulation were correlated, as older children are likely better able to self-regulate. Finally, the errors between supportive coparenting and father involvement at Wave 1 were correlated, as fathers’ involvement is greater when supportive coparenting is high (Fagan and Palkovitz, 2019). In line with recommendations to avoid listwise deletion, the variances for remaining ordinal predictors were estimated to address missing data via FIML (Muthén and Muthén, 2017).

Finally, a multi-group path analysis was conducted to determine whether there were differences in the associations between fathers’ parenting quality and children’s self-regulation by fathers’ residential status. Cross-group invariance was assessed by comparing two nested models: (1) a baseline model wherein no constraints are specified (i.e., all parameters are freely estimated) and (2) a second model where the paths of variables of interest are constrained to be equal. A Satorra-Bentler chi-square difference test was used to determine if differences between models were statistically significant.

Several fit indices were used to determine the extent to which the hypothesized model was an adequate fit for the data. Namely, a chi-square test was used to determine model fit, with a non-significant chi-square test indicating acceptable fit. Additionally, the absolute fit of the model was examined using a cutoff of 0.06 the root mean square error of approximation (RMSEA; McDonald and Ho, 2002). Additionally, for comparative fit indices (CFI), a cutoff of 0.95 was considered acceptable (Hu and Bentler, 1999).

## Results

### Sample Characteristics

Of all eligible fathers who participated in the EDS (*n* = 257), 245 fathers reported information on the focal child’s gender. Of these fathers, 139 reported having a male child and 106 reported having a female child. On average, children who participated in Wave 1 of the EDS subsample were age 3.15 years (*SD* = 0.91) and children who participated in Wave 2 of the EDS subsample were age 4.38 years (*SD* = 0.91).

Participating fathers were, on average, 30.2 years of age at Wave 1 (*SD* = 7.54; min = 18 years; max = 53 years). Approximately 45.1% identified as Hispanic, 44.4% identified as non-Hispanic Black or African American, 7.8% of fathers identified as White, and 2.7% identified as non-Hispanic, other. Seventy-seven percent of fathers were born in the United States. Interviews were conducted in Spanish and English. Approximately 63.5% of fathers reported that they were never married, 29.5% percent of fathers reported that they were currently married to the focal child’s biological mother, and approximately 7% of fathers reported that they had married the focal child’s biological mother at some point but were now separated or divorced. Approximately 31.1% of fathers reported having a high school diploma, 28% reported no diploma, certification, or degree, 24.5% reported a high school equivalency diploma, 7% reported having a vocational tech diploma, 5.4% reported having an associate’s degree, and 3.9% of fathers reported holding a bachelor’s degree or higher. Total number of usual hours worked per week across all jobs ranged from 1 h to 96 h per week. On average, fathers reported working 41.7 h per week (*SD* = 15.48). Fathers reported that, on average, their income from all sources last month was $983 (*SD* = 1204.44).

### Preliminary Analysis

Table 1 includes means, standard deviations, and descriptive statistics for all variables of interest grouped by all fathers, resident fathers, and non-resident fathers. On average, children of resident fathers did not have statistically significantly different levels of self-regulation from children of non-resident fathers in the gift wrap [*t*(189) = 0.13, *p* = 0.90] or snack delay tasks [*t*(189) = −0.04, *p* = 0.97] at Wave 2. Intercorrelations among key variables of interest are reported in Table 2. Of note, fathers’ authoritative parenting behavior was statistically significantly associated with greater levels of self-regulation in the gift wrap (*r* = 0.17, *p* < 0.05) and snack delay tasks (*r* = 0.17, *p* < 0.05) at Wave 1. Fathers’ reported hours of involvement at Wave 1 were associated with greater self-regulation in the gift wrap task at Wave 2 at a level that was approaching significance (*r* = 0.14, *p* < 0.10). Coparenting support at Wave 1 was positively associated with children’s self-regulation in the gift wrap task at Wave 2 at a level approaching significance (*r* = 0.13, *p* < 0.10). As expected, older children exhibited greater self-regulation in the gift wrap task (*r* = 0.36, *p* < 0.01) and snack delay task (*r* = 0.43, *p* < 0.01) at Wave 2.

**TABLE 1:** Means and descriptive statistics of father involvement and child self-regulation by fathers’ residential status.

		All fathers			Resident fathers			Non-resident fathers		
	Range	*N*	*M*	*SD*	*N*	*M*	*SD*	*N*	*M*	*SD*
**Wave 1**										
**Self-regulation (observed)**										
Snack delay	−1.80 – 1.13	218	−0.04	0.94	95	−0.08	0.97	123	−0.01	0.92
Gift wrap	−1.30 – 1.26	214	−0.01	0.92	93	0.05	0.89	121	−0.06	0.94
**Father involvement (father reported)**										
Authoritative parenting	2.0 – 4.0	251	3.44	0.41	105	3.39	0.44	146	3.48	0.39
Harsh parenting	1.0 – 4.0	254	2.54	1.06	106	2.59	1.06	148	2.50	1.06
Hours of involvement	0.0 – 168.0	237	38.62	40.61	98	56.86	44.77	139	25.76	31.76
**Mother involvement (mother reported**										
Authoritative parenting	1.86 – 4.00	250	3.54	0.41	104	3.59	0.37	146	3.51	0.43
Harsh parenting	1.00 – 4.00	249	2.73	1.08	101	2.71	1.13	148	2.74	1.06
**Coparenting**										
Support	1.0 – 4.0	236	3.23	0.85	105	3.70	0.43	131	2.86	0.92
**Wave 2**										
**Self-regulation (observed)**										
Snack delay	−3.86 – 0.84	191	−0.04	0.91	79	−0.04	0.93	112	−0.04	0.90
Gift wrap	−2.51 – 0.72	191	−0.05	0.92	79	−0.04	0.90	112	−0.06	0.95

*Fathers’ hours of involvement were standardized in statistical analyses. However, the unstandardized hours are depicted here to facilitate interpretability.*

**TABLE 2:** Intercorrelations among study variables of interest.

Wave 1	1.	2.	3.	4.	5.	6.	7.	8.	9.	10.	11.
1. Gift wrap task	–										
2. Snack delay task	0.578**	–									
3. Authoritative parenting (F)	0.173*	0.168*	–								
4. Harsh parenting (F)	0.007	−0.012	0.236**	–							
5. Authoritative parenting (M)	0.138*	0.184**	0.129*	−0.030	–						
6. Harsh parenting (M)	−0.032	0.021	0.098	0.334**	0.014	–					
7. Hours of involvement	0.024	0.088	0.144*	0.200*	−0.014	0.097	–				
8. Support	0.037	0.059	−0.025	0.062	0.078	−0.047	0.300**	–			
**Wave 2**											
9. Child age	0.476**	0.564**	0.280**	0.014	0.365**	0.066	0.081	0.031	–		
10. Gift wrap task	0.285**	0.343**	0.118	−0.023	0.106	−0.054	0.142^+^	0.132^+^	0.360**	–	
11. Snack delay task	0.244**	0.281**	0.018	−0.089	0.214**	−0.049	−0.111	−0.013	0.425**	0.350**	–

^+^p < 0.10; *p < 0.05; **p < 0.01.

*F = Father and M = Mother.*

### Path Analyses Predicting Children’s Self-Regulation at Wave 2 From Fathers’ Quality of Involvement, Quantity of Involvement, and Coparenting at Wave 1

In the second stage of the analysis, fathers’ authoritative parenting, harsh parenting, hours of involvement, and coparenting support were included as predictors of children’s self-regulation at Wave 2, while controlling for child self-regulation and mothers’ parenting (i.e., authoritative and harsh parenting) at Wave 1. Child gender, age, and fathers’ education level were also included as control variables. Fit indices indicated that the model fit the data well [χ^2^(12) = 13.97, *p* = 0.30; CFI = 0.979; RMSEA = 0.026].

Fathers’ quantity of involvement positively predicted child self-regulation in the gift wrap task (β = 0.11, *p* < 0.05). Supportive coparenting and fathers’ reports of parenting quality did not emerge as statistically significant predictors in either task. Associations among control variables and children’s self-regulation were observed. Namely, greater self-regulation in the gift wrap task at Wave 1 was associated with greater self-regulation in the gift wrap task at Wave 2 at a level approaching significance (β = 0.15, *p* = 0.066). Older children exhibited greater levels of self-regulation in the gift wrap task (β = 0.28, *p* < 0.001) and snack delay (β = 0.44, *p* < 0.001) tasks at Wave 2. Female children exhibited greater self-regulation in the snack delay task (β = 0.24, *p* < 0.001). Fathers’ education level did not statistically significantly predict child self-regulation in the gift wrap task (β = 0.06, *p* = 0.36). However, fathers’ education level was positively associated with self-regulation in the snack delay task at a level approaching significance (β = 0.12, *p* = 0.053).

### Examining Differences by Fathers’ Residential Status

In the third stage of the analysis, multi-group path analyses were conducted to evaluate whether the model fit the data equally well for resident and non-resident fathers. The freely estimated model had acceptable fit [χ^2^(29) = 34.15, *p* = 0.23; CFI = 0.95; RMSEA 0.038]. Next, structural paths of interest were constrained to be equal between resident and non-resident fathers [χ^2^(44) = 59.70, *p* = 0.057; CFI = 0.85; RMSEA 0.054]. A Satorra–Bentler chi-square difference test indicated that constraining the structural paths across resident and non-resident fathers resulted in statistically significantly worsening the overall fit of the model [Δχ^2^(15) = 26.21, *p* = 0.0358], rejecting the null hypothesis that the paths (on the whole) were the same for resident and non-resident fathers. Thus, patterns of association were statistically significantly different between resident and non-resident fathers.

**FIGURE 1:**
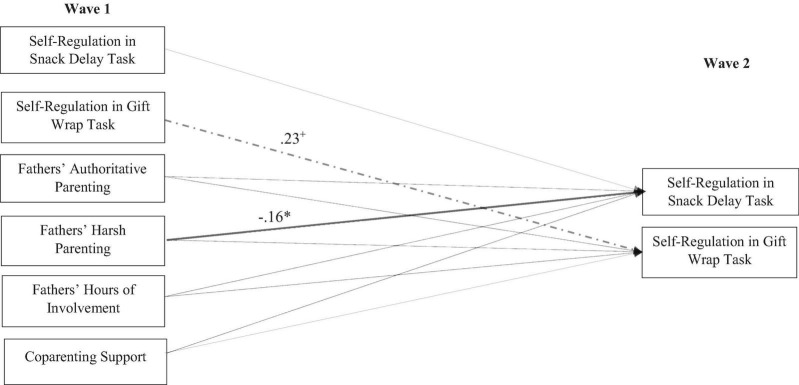
Associations between resident fathers’ parenting, coparenting, and children’s self-regulation at Wave 1 and Wave 2. Child age, mothers’ parenting (i.e., authoritative and harsh), child gender, and father education level were included as control variables but (to more effectively show associations among paths of interest) were not included in the above figure. Statistically significant estimates are depicted in solid bold lines. Estimates that are statistically significant at trend-level are depicted in the dashed line. Dotted lines indicate estimates that were not statistically significant. χ^2^(29) = 34.15, *p* = 0.23; CFI = 0.950; RMSEA = 0.038; *p < 0.05; ^+^*p* < 0.10.

As depicted in Figure 1, resident fathers’ harsh parenting at Wave 1 statistically significantly predicted lower levels of self-regulation in the snack delay task at Wave 2 (β = −0.16, *p* < 0.05). Supportive coparenting was not statistically significantly associated with child self-regulation in the snack delay (β = 0.09, *p* = 0.32) or gift wrap (β = −0.04, *p* = 0.65) tasks. Additionally, resident fathers’ quantity of involvement was not associated with child self-regulation in the snack delay (β = −0.12, *p* = 0.27) or gift wrap tasks (β = 0.05, *p* = 0.53). Child age remained a statistically significant predictor of child self-regulation in the snack delay (β = 0.38, *p* < 0.01) and gift wrap tasks (β = 0.22, *p* < 0.05). Female children exhibited greater self-regulation in the snack delay task (β = 0.35, *p* < 0.01). Fathers’ education level did not emerge as a statistically significant predictor of child self-regulation in either task.

**FIGURE 2:**
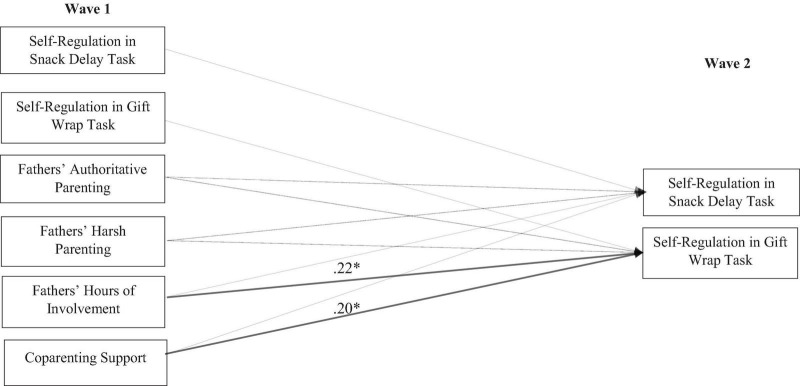
Associations between non-resident fathers’ parenting, coparenting, and children’s self-regulation at Wave 1 and Wave 2. Child age, mothers’ parenting (i.e., authoritative and harsh), child gender, and father education level were included as control variables but (to more effectively show associations among paths of interest) were not included in the above figure. Statistically significant estimates are depicted in solid bold lines. Dotted lines indicate estimates that were not statistically significant. χ^2^(29) = 34.15, *p* = 0.23; CFI = 0.950; RMSEA = 0.038; *p < 0.05.

As depicted in Figure 2, non-resident fathers’ reported hours of involvement at Wave 1 statistically significantly predicted greater child self-regulation in the gift wrap task at Wave 2 (β = 0.22, *p* < 0.05). Additionally, supportive coparenting at Wave 1 predicted greater child self-regulation in the gift wrap task at Wave 2 (β = 0.20, *p* < 0.05). Child age remained a statistically significant predictor of child self-regulation in the snack delay (β = 0.52, *p* < 0.01) and gift wrap tasks (β = 0.37, *p* < 0.01). Female children exhibited greater self-regulation in the snack delay task (β = 0.14, *p* < 0.05) and the gift wrap task at a level approaching significance (β = 0.16, *p* = 0.053). Fathers’ education level predicted greater child self-regulation in the snack delay task (β = 0.19, *p* < 0.05) but not the gift wrap task.

## Discussion

The development of children’s self-regulation, occurring from birth through children’s early preschool and elementary years, has significant implications for subsequent adjustment, including better academic achievement and peer relationships in middle childhood and beyond. Scientists have made significant strides in understanding associations between mothers’ parenting and child self-regulation in recent years. However, surprisingly few studies have examined the role of coparenting and fathers’ parenting (Roggman et al., 2013). Furthermore, when study resources are limited, low-income and non-resident fathers are often overlooked due to the difficulty of recruiting, tracking, and following-up. The primary purpose of this study was to investigate fathers’ parenting quality (i.e., authoritative and harsh parenting behaviors), quantity of involvement, and supportive coparenting as predictors of children’s self-regulation in a sample of families facing economic hardship.

Fathers’ parenting quality and quantity of involvement predicted greater child self-regulation. However, the associations between fathers’ reports of parenting quality (i.e., authoritative and harsh parenting), quantity of involvement, and children’s self-regulation varied by fathers’ residential status. For non-resident fathers, authoritative and harsh parenting were not linked to children’s self-regulation. In contrast, reports of fathers’ quantity of involvement were positively associated with better self-regulation in children. This finding was somewhat unexpected, as prior meta-analyses have indicated non-resident fathers’ quality of involvement is more closely tied to positive child outcomes than quantity of father involvement (Adamsons and Johnson, 2013). However, this study’s unique aspects may contextualize this difference. First, this study relied on fathers’ perceptions of their involvement, whereas most studies rely on mothers’ perceptions of non-resident father involvement. Second, this is the first study (to the author’s knowledge) to consider longitudinal associations between non-resident fathers’ perceptions of their involvement and observed child self-regulation. Perhaps non-resident fathers’ quantity of involvement is a more salient predictor of child self-regulation than other developmental outcomes (i.e., academic performance and social-emotional adjustment). Nevertheless, this finding aligns with the view that non-resident fathers’ quantity of involvement is important to consider when promoting positive child outcomes (Adamsons, 2018).

How involved were non-resident fathers? On average, non-resident fathers reported 25.76 h of involvement per week in the current study. However, it is difficult to directly compare the hours of involvement reported in this study and other studies of non-resident father involvement due to differential question phrasing and respondents. Studies examining father involvement among low-income, non-resident United States fathers (i.e., Choi et al., 2014) have typically relied on mothers’ reports of non-resident fathers’ frequency of contact with the child. Information about non-resident father involvement are often obtained via ordinal surveys or open-ended questions over a longer duration of time (i.e., “How many days has father seen child during the past 30 days?”). Thus, it is challenging to make direct comparisons between non-resident father involvement in this study and prior research. As families become increasingly diverse, researchers should more thoroughly examine non-resident father involvement across various contexts.

Findings also indicated, for resident fathers, harsh parenting behaviors were longitudinally associated with decreased self-regulation in children. Resident fathers’ quantity of involvement, in contrast, was not statistically significantly associated with children’s self-regulation. Although this study is unique in its focus on low-income, biological fathers, this finding is consistent with other research indicating that greater harsh parenting among adoptive fathers was associated with lower child self-regulation (Bridgett et al., 2018). In longitudinal research that has focused on mothers’ parenting, greater maternal warmth and low levels of physically punitive discipline emerged as predictors of children’s greater capacity for self-regulation in middle childhood (Colman et al., 2006).

It is important to note that the parenting behaviors included in the harsh parenting measure focused exclusively on spanking behavior. On average, parents in the United States report spanking at higher rates than parents in other industrialized nations. Spanking, in turn, has predicted greater social-emotional difficulties in early childhood (Pace et al., 2019). Fathers’ spanking, in particular, has been linked to increased aggression in preschool-aged children (Lee et al., 2013). Furthermore, prior research using data from the Family Life Project (i.e., low-income, rural children) found that fathers’ negativity was more closely tied to child stress regulation than positive parenting behaviors (Mills-Koonce et al., 2011). Thus, this study contributes to a growing body of research highlighting the negative consequences of fathers’ harsh parenting behaviors.

It may also be important to consider the context in which harsh parenting behavior is delivered. For example, researchers have suggested that the consequences of harsh parenting for child maladjustment depend on whether discipline is delivered in an emotionally charged or controlled manner (Chang et al., 2003). Thus, more detailed information on the nature in which fathers’ harsh parenting is delivered might provide further insight into its role in the development of children’s self-regulation. Additionally, when multiple harsh parenting behaviors co-occur, children may be most at risk for self-regulation difficulties (see Mills-Koonce et al., 2016).

Notably, the reported associations between fathers’ quantity and quality of involvement were statistically significant even when controlling for mothers’ parenting quality and earlier levels of child self-regulation. Thus, these data would suggest that, for children with a non-resident father, fathers’ quantity of involvement is important for developing children’s self-regulation. Additionally, harsh parenting may be particularly detrimental when fathers live with the child. However, findings may not generalize to all fathers. That is, *any* type of involvement is not necessarily beneficial for the development of children’s self-regulation. In some cases, mothers report engaging in gatekeeping behavior, or attempting to discourage and limit fathers’ opportunity for involvement in childrearing, because fathers pose a threat to the child’s health and well-being (Zvara et al., 2016).

Finally, this study was among the first (to the author’s knowledge) to examine whether associations between supportive coparenting and child self-regulation varied by fathers’ residential status. Supportive coparenting emerged as a predictor of child self-regulation among children with a non-resident father. However, supportive coparenting did not predict child self-regulation among children with a resident father. Thus, in line with the emotional security hypothesis (Davies et al., 2002), supportive coparenting may be especially important in contributing to a positive emotional climate in the home when fathers are non-resident. Perhaps in these situations parents who have positive coparenting relationships can set aside personal disagreements and differences and prioritize taking a team-oriented approach to childrearing. As a result, parents who support each other’s parenting strategy can cultivate a calm and respectful atmosphere. This favorable climate supports children’s sense of security and emerging self-regulation skills.

Importantly, this study focused exclusively on resident and non-resident United States fathers facing economic hardship. In the United States, the percentage of children living with two parents versus a single parent varies dramatically based on family socioeconomic status, with a greater proportion of non-resident fathers among lower socioeconomic status families. Therefore, study findings should be interpreted cautiously when considering how they might generalize to higher socioeconomic statuses. An emerging area of research has examined whether the effects of non-resident father involvement are stronger for children in low-SES households compared to high-SES households. Results have indicated that non-resident father involvement was similarly positive for child outcomes regardless of family SES (Tanskanen and Erola, 2017; Miller et al., 2020). Thus, it is expected that non-resident father involvement would be similarly beneficial for children in higher-SES families.

Although this study provides important insight into resident and non-resident fathers’ parenting and children’s self-regulation, study limitations should be addressed. The non-resident fathers who agreed to participate in this study were, on the whole, more involved in their children’s lives than fathers who declined participation. In addition, mothers provided contact information for non-resident fathers. Thus, it is likely that the coparenting relationship between parents was stronger in cases where mothers provided contact information and fathers agreed to participate, compared to situations in which mothers refused to give the researchers fathers’ contact information. Indeed, in some cases, mothers refused to provide contact information because fathers were in prison, mothers were afraid fathers would be mad at them, or mothers did not want fathers to be involved in any part of the child’s life. Additionally, in the United States, there are various types of non-resident fathers (including non-resident fathers who live out of state). Future research is needed to determine how non-resident fathers who see their child infrequently, but use technology to stay in touch, may contribute to child self-regulation.

A second limitation is that the reliabilities for fathers’ perceptions of their parenting quality (i.e., authoritative and harsh parenting) were lower than for mothers’ perceptions. In studies that include both mothers’ and fathers’ parenting measures, it is common to see lower reliability among fathers. This discrepancy may occur because researchers apply measures that have been developed and validated on mothers to fathers (Roggman et al., 2012). Many of the parenting measures were built around conceptions of mothers’ parenting—often referred to as the “maternal template” (Marsiglio et al., 2000). Family systems researchers primarily rely on measures originally developed on mothers to assess fathers’ parenting because this approach enables a more direct comparison between mothers and fathers (Fagan et al., 2014).

Third, the measure of father involvement assessed fathers’ perceptions of their quantity of involvement via a single question. In general, time diaries are considered a more thorough method for assessing involvement. However, notwithstanding this limitation, this study is unique in its inclusion of fathers’ perceptions of their *own* quantity of involvement. Thus, this study expands upon existing research (i.e., Choi et al., 2014), which has relied more often on mothers’ reports of non-resident father involvement.

There are several avenues for future research. Although several parenting programs focus on building positive relationships among non-resident fathers and their children, efforts targeted at improving the measurement of non-resident fathers’ parenting have lagged. Researchers are only just beginning to develop and validate measures of parenting on non-resident fathers (i.e., Dyer et al., 2018). Better assessing the nature of non-resident father involvement is necessary for informing parenting programming and intervention efforts. Additionally, non-resident fathers may contribute to child outcomes through other pathways, such as child support payments. When fathers are experiencing economic hardship, it may be especially challenging to comply with child support arrangements, which may lead to conflict in the coparenting relationship.

Finally, father figures—including stepfathers and maternal boyfriends—may contribute to children’s self-regulation. Although there were some stepfather and boyfriend father figures identified at Wave 1, the sample size was too small to make meaningful comparisons between children with a stepfather or maternal boyfriend father figure and children without one. Future research focusing more specifically on the role of father figures and non-resident fathers to the development of young children’s self-regulation may yield important insights.

Despite some limitations, this study supports increasing interest in policies and programs that promote father involvement. In particular, one way to support non-resident father involvement may be to increase the availability of paternity leave. Paternity leave may provide an opportunity for fathers to develop a secure attachment bond, establish a routine with their baby, and develop a strong coparenting foundation. Non-resident fathers who take paternity leave are more involved and more likely to look after the child when the mother needs assistance (Knoester et al., 2019; Pilkauskas and Schneider, 2020).

Furthermore, several educational efforts may help practitioners and clinicians reduce harsh discipline practices among parents. For example, showing parents research findings on the adverse effects of spanking reduces the view that spanking is an appropriate discipline strategy (Holden et al., 2014). In addition, pediatricians are often trusted sources for parents. Therefore, providing brief education in waiting rooms regarding the consequences of harsh discipline strategies may prove beneficial.

In sum, this study contributes to emerging research examining associations between fathers’ parenting quality and quantity of involvement, coparenting, and children’s subsequent self-regulation. The development of children’s self-regulation, occurring from birth through children’s early preschool and elementary years, has significant implications for subsequent adjustment, including better academic achievement and peer relationships during middle childhood and beyond (Blair and Razza, 2007). By controlling for mothers’ parenting quality and children’s earlier self-regulation, this study offers insights into what aspects of the family system best support child self-regulation—especially in the context of economic hardship.

## Data Availability Statement

Publicly available datasets were analyzed in this study. This data can be found here: https://www.icpsr.umich.edu/web/DSDR/studies/4701/.

## Ethics Statement

The studies involving human participants were reviewed and approved by Penn State University (PSU). PSU determined that the study does not meet the definition of human subjects research as the data were not restricted, deidentified, and no code list was shared (STUDY0018723). Written informed consent to participate in this study was provided by the participants’ legal guardian/next of kin.

## Author Contributions

This manuscript and its contents are solely the responsibility of LA and do not necessarily represent the official views of The Pennsylvania State University.

## Conflict of Interest

The author declares that the research was conducted in the absence of any commercial or financial relationships that could be construed as a potential conflict of interest.

## Publisher’s Note

All claims expressed in this article are solely those of the authors and do not necessarily represent those of their affiliated organizations, or those of the publisher, the editors and the reviewers. Any product that may be evaluated in this article, or claim that may be made by its manufacturer, is not guaranteed or endorsed by the publisher.

## Footnotes

1 Traditional methods to estimate missing data (i.e., FIML) assume that the data are missing completely at random or missing at random. The eight participants that were omitted from the analysis were not asked questions on key variables of interest (i.e., parenting) due to a lack of contact with their child.

## References

[B1] Aber J. L. (2012). “Poor and low-income families, infant/toddler development and the prospects for change: Back to the future” in *Infants, Toddlers, and Families in Poverty: Research Implications for Early Child Care.* eds Odom S. L. Pungello E. Gardner-Neblett N. (United States: Guilford Press). 3–18.

[B2] Adamsons K. (2018). Quantity versus quality of nonresident father involvement: Deconstructing the argument that quantity doesn’t matter. *J. Child Custody* 15 26–34. 10.1080/15379418.2018.1437002

[B3] Adamsons K. Johnson S. K. (2013). An updated and expanded meta-analysis of nonresident fathering and child well-being. *J. Fam. Psychol.* 27 589–599. 10.1037/a0033786 23978321

[B4] Angel R. Burton L. Chase-Lansdale P. L. Cherlin A. Moffitt R. (2012). *Welfare, Children, and Families: A Three-City Study.* Ann Arbor, MI: Inter-University Consortium for Political and Social Research.

[B5] Baptista J. Sousa D. Soares I. Martins C. (2018). Fathers’ sensitive guidance moderates the association between coparenting and behavioral regulation in preschoolers. *Int. J. Behav. Dev.* 42 574–580. 10.1177/0165025418761816

[B6] Bernier A. Carlson S. M. Deschênes M. Matte-Gagné C. (2012). Social factors in the development of early executive functioning: A closer look at the caregiving environment. *Dev. Sci.* 15 12–24. 10.1111/j.1467-7687.2011.01093.x 22251288

[B7] Bernier A. Carlson S. M. Whipple N. (2010). From external regulation to self-regulation: Early parenting precursors of young children’s executive functioning. *Child Dev.* 81 326–339. 10.1111/j.1467-8624.2009.01397.x 20331670

[B8] Blair C. (2010). Stress and the development of self-regulation in context. *Child Dev. Perspect.* 4 181–188. 10.1111/j.1750-8606.2010.00145.x 21779305 PMC3138186

[B9] Blair C. Razza R. P. (2007). Relating effortful control, executive function, and false belief understanding to emerging math and literacy ability in kindergarten. *Child Dev.* 78 647–663. 10.1111/j.1467-8624.2007.01019.x 17381795

[B10] Bocknek E. L. Dayton C. Raveau H. A. Richardson P. Brophy-Herb H. E. Fitzgerald H. E. (2017). Routine active playtime with fathers is associated with self-regulation in early childhood. *Merrill Palmer Q.* 63 105–134. 10.13110/merrpalmquar1982.63.1.0105

[B11] Bridgett D. J. Ganiban J. M. Neiderhiser J. M. Natsuaki M. N. Shaw D. S. Reiss D. (2018). Contributions of mothers’ and fathers’ parenting to children’s self-regulation: Evidence from an adoption study. *Dev. Sci.* 21:e12692. 10.1111/desc.12692 29978935 PMC6202135

[B12] Brophy-Herb H. E. Stansbury K. Bocknek E. Horodynski M. A. (2012). Modeling maternal emotion-related socialization behaviors in a low-income sample: Relations with toddlers’ self-regulation. *Early Childhood Res. Q.* 27 352–364. 10.1016/j.ecresq.2011.11.005

[B13] Calkins S. D. (2007). “The emergence of self-regulation: Biological and behavioral control mechanisms supporting toddler competences” in *Socioemotional Development in the Toddler Years: transitions and Transformations.* eds Brownell C. A. Kopp C. B. (United States: Guilford). 261–284. 10.4324/9781003086062-9

[B14] Carlson M. J. (2006). Family structure, father involvement, and adolescent behavioral outcomes. *J. Marr. Fam.* 68 137–154. 10.1111/j.1741-3737.2006.00239.x

[B15] Carlson M. J. McLanahan S. S. (2010). “Fathers in fragile families” in *The Role of the Father in Child Development.* ed. Lamb M. E. (United States: John Wiley & Sons, Inc). 10.4324/9780203792292-8

[B16] Carver C. S. Scheier M. F. (2016). “Self-regulation of action and affect” in *Handbook of Self-regulation: research, Theory, and Applications.* 3rd Edn, eds Vohs K. D. Baumeister R. F. (United States: Guilford Press).

[B17] Census (2016). *The Majority of Children Live with Two Parents: census Data Reports.* Available online at: https://www.census.gov/newsroom/press-releases/2016/cb16-192.html (accessed August, 2021)

[B18] Census (2020). Living Arrangements of Children Under 18 Years and Marital Status of Parents, by Age, Sex, Race, and Hispanic Origin and Selected Characteristics of the Child for All Children: 2020. Available online at: https://www.census.gov/data/tables/2020/demo/families/cps-2020.html (accessed December, 2021)

[B19] Chang L. Schwartz D. Dodge K. A. McBride-Chang C. (2003). Harsh parenting in relation to child emotion regulation and aggression. *J. Fam. Psychol.* 17:598. 10.1037/0893-3200.17.4.598 14640808 PMC2754179

[B20] Choe D. E. Olson S. L. Sameroff A. J. (2013). Effects of early maternal distress and parenting on the development of children’s self-regulation and externalizing behavior. *Dev. Psychopathol.* 25 437–453. 10.1017/S0954579412001162 23627955

[B21] Choi J. K. Palmer R. J. Pyun H. S. (2014). Three measures of non-resident fathers’ involvement, maternal parenting and child development in low-income single-mother families. *J. Fam. Soc. Work* 19 282–291. 10.1111/cfs.12000

[B22] Ciairano S. Visu-Petra L. Settanni M. (2007). Executive inhibitory control and cooperative behavior during early school years: A follow-up study. *J. Abnorm. Child Psychol.* 35 335–345. 10.1007/s10802-006-9094-z 17226093

[B23] Colman R. A. Hardy S. A. Albert M. Raffaelli M. Crockett L. (2006). Early predictors of self-regulation in middle childhood. *Infant Child Dev.* 15 421–437. 10.1002/icd.469

[B24] Cox M. J. Paley B. (2003). Understanding families as systems. *Curr. Dir. Psychol. Sci.* 12 193–196. 10.1111/1467-8721.01259

[B25] Davies P. T. Harold G. T. Goeke-Morey M. C. Cummings E. M. Shelton K. Rasi J. A. (2002). Child emotional security and interparental conflict. *Monogr. Soc. Res. Child Dev.* 67 i–v, vii–viii, 1–115. 12528424

[B26] Diamond A. Kirkham N. Amso D. (2002). Conditions under which young children can hold two rules in mind and inhibit a prepotent response. *Dev. Psychol.* 38 352–362. 10.1037/0012-1649.38.3.352 12005379

[B27] Drake K. Belsky J. Fearon R. (2014). From early attachment to engagement with learning in school: The role of self-regulation and persistence. *Dev. Psychol.* 50 1350–1361. 10.1037/a0032779 23647414

[B28] Dyer W. J. Kauffman R. Fagan J. Pearson J. Cabrera N. (2018). Measures of father engagement for nonresident fathers. *Fam. Rel.* 67 381–398. 10.1111/fare.12317

[B29] Eisenberg N. Valiente C. Eggum N. D. (2010). Self-regulation and school readiness. *Early Educ. Dev.* 21 681–698. 10.1080/10409289.2010.497451 21234283 PMC3018834

[B30] Fagan J. Day R. Lamb M. E. Cabrera N. J. (2014). Should researchers conceptualize differently the dimensions of parenting for fathers and mothers? *J. Fam. Theory Rev.* 6 390–405. 10.1111/jftr.12044

[B31] Fagan J. Palkovitz R. (2019). Coparenting and father engagement among low-income parents: Actor–partner interdependence model. *J. Fam. Psychol.* 33 894–904. 10.1037/fam0000563 31318267

[B32] Farr R. H. Bruun S. T. Patterson C. J. (2019). Longitudinal associations between coparenting and child adjustment among lesbian, gay, and heterosexual adoptive parent families. *Dev. Psychol.* 55 2547–2560. 10.1037/dev0000828 31512896

[B33] Feinberg M. E. (2003). The internal structure and ecological context of coparenting: A framework for research and intervention. *Parent. Sci. Pract.* 3 95–131. 10.1207/S15327922PAR0302_01 21980259 PMC3185375

[B34] Gagne J. R. Saudino K. J. (2016). The development of inhibitory control in early childhood: A twin study from 2–3 years. *Dev. Psychol.* 52 391–399. 10.1037/dev0000090 26784384 PMC4839189

[B35] Gray P. B. Brown E. (2015). Fatherhood in St. Kitts: Patterns and Predictors of Partnership and Paternal Dynamics in a Caribbean Island. *Father. J. Theory Res. Pract. Men Fathers* 13:18-35. 10.3149/fth.1301.18

[B36] Grossmann K. Grossmann K. E. Fremmer-Bombik E. Kindler H. Scheuerer-Englisch H. Zimmermann (2002). The uniqueness of the child–father attachment relationship: Fathers’ sensitive and challenging play as a pivotal variable in a 16-year longitudinal study. *Soc. Dev.* 11 301–337. 10.1111/1467-9507.00202

[B37] Gunnar M. R. Fisher P. A. (2006). Bringing basic research on early experience and stress neurobiology to bear on preventive interventions for neglected and maltreated children. *Dev. Psychopathol.* 18 651–677. 10.1017/S0954579406060330 17152395

[B38] Hewlett B. S. Lamb M. E. Shannon D. Leyendecker B. Schölmerich A. (1998). Culture and early infancy among central African foragers and farmers. *Dev. Psychol.* 34 653–661. 10.1037/0012-1649.34.4.653 9681257

[B39] Holden G. W. Brown A. S. Baldwin A. S. Caderao K. C. (2014). Research findings can change attitudes about corporal punishment. *Child Abuse Negl.* 38 902–908. 10.1016/j.chiabu.2013.10.013 24246718

[B40] Holmboe K. Fearon R. P. Csibra G. Tucker L. A. Johnson M. H. (2008). Freeze-Frame: A new infant inhibition task and its relation to frontal cortex tasks during infancy and early childhood. *J. Exp. Child Psychol.* 100 89–114. 10.1016/j.jecp.2007.09.004 18249410

[B41] Hu L. T. Bentler P. M. (1999). Cutoff criteria for fit indexes in covariance structure analysis: Conventional criteria versus new alternatives. *Struct. Equat. Model. Multidiscipl. J* 6 1–55. 10.1080/10705519909540118

[B42] Jenson J. M. Fraser M. W. (2016). “A risk and resilience framework for child, youth, and family policy” in *Social Policy for Children and Families: a Risk and Resilience Perspective.* eds Jenson J. M. Fraser M. W. (United States: Sage). 5–21.

[B43] Julian M. M. Leung C. Y. Rosenblum K. L. LeBourgeois M. K. Lumeng J. C. Kaciroti N. (2019). Parenting and toddler self-regulation in low-income families: What does sleep have to do with it? *Infant Ment. Health J.* 40 479–495. 10.1002/imhj.21783 31066463 PMC6842328

[B44] Knoester C. Petts R. J. Pragg B. (2019). Paternity leave-taking and father involvement among socioeconomically disadvantaged US fathers. *Sex Roles* 81 257–271. 10.1007/s11199-018-0994-5 31406394 PMC6690499

[B45] Kochanska G. Murray K. Jacques T. Y. Koenig A. L. Vandegeest K. A. (1996). Inhibitory control in young children and its role in emerging internalization. *Child Dev.* 67 490–507. 10.1111/j.1467-8624.1996.tb01747.x 8625724

[B46] Kuhlman K. R. Repetti R. L. Reynolds B. M. Robles T. F. (2018). Interparental conflict and child HPA-axis responses to acute stress: Insights using intensive repeated measures. *J. Fam. Psychol.* 32 773–782. 10.1037/fam0000437 29927288 PMC6126984

[B47] Lamb M. E. Pleck J. H. Charnov E. L. Levine J. A. (1985). Paternal behavior in humans. *Am. Zool.* 25 883–894. 10.4103/0022-3859.186389 27424551 PMC4970346

[B48] Lamb M. E. Pleck J. H. Charnov E. L. Levine J. A. (1987). “A biosocial perspective on paternal behavior and involvement” in *Parenting Across the Lifespan: biosocial Dimensions.* eds Lancaster J. B. Altmann J. Rossi A. S. Sherrod L. R. (Berlin: Aldine de Gruyter). 111–142.

[B49] Lee S. J. Taylor C. A. Altschul I. Rice J. C. (2013). Parental spanking and subsequent risk for child aggression in father-involved families of young children. *Child. Youth Serv. Rev.* 35 1476–1485. 10.1016/j.childyouth.2013.05.016 24019558 PMC3765035

[B50] Little T. D. Jorgensen T. D. Lang K. M. Moore E. W. G. (2014). On the joys of missing data. *J. Pediatr. Psychol.* 39 151–162. 10.1093/jpepsy/jst048 23836191

[B51] Lucassen N. Kok R. Bakermans-Kranenburg M. J. Van Ijzendoorn M. H. Jaddoe V. W. Hofman A. (2015). Executive functions in early childhood: The role of maternal and paternal parenting practices. *Br. J. Dev. Psychol.* 33 489–505. 10.1111/bjdp.12112 26359942

[B52] Macdonald J. A. Beauchamp M. H. Crigan J. A. Anderson P. J. (2014). Age-related differences in inhibitory control in the early school years. *Child Neuropsychol.* 20 509–526. 10.1080/09297049.2013.822060 23909718

[B53] Marsiglio W. Amato P. Day R. D. Lamb M. E. (2000). Scholarship on fatherhood in the 1990s and beyond. *J. Marr. Fam.* 62 1173–1191. 10.1111/j.1741-3737.2000.01173.x

[B54] Matthews J. S. Ponitz C. C. Morrison F. J. (2009). Early gender differences in self-regulation and academic achievement. *J. Educ. Psychol.* 101:689. 10.1037/a0014240

[B55] McDonald R. P. Ho M.-H. R. (2002). Principles and practice in reporting structural equation analyses. *Psychol. Methods*, 7 64–82. 10.1037/1082-989X.7.1.64 11928891

[B56] Meece D. Robinson C. M. (2014). Father–child interaction: associations with self-control and aggression among 4.5-year-olds. *Early Child Dev. Care* 184 783–794. 10.1080/03004430.2013.818990

[B57] Miller D. P. Thomas M. M. Waller M. R. Nepomnyaschy L. Emory A. D. (2020). Father involvement and socioeconomic disparities in child academic outcomes. *J. Marr. Fam.* 82 515–533. 10.1111/jomf.12666

[B58] Mills-Koonce W. R. Garrett-Peters P. Barnett M. Granger D. A. Blair C. Cox M. J. (2011). Father contributions to cortisol responses in infancy and toddlerhood. *Dev. Psychol.* 47:388. 10.1037/a0021066 21142362 PMC4428321

[B59] Mills-Koonce W. R. Willoughby M. T. Garrett-Peters P. Wagner N. Vernon-Feagans L. (2016). The interplay among socioeconomic status, household chaos, and parenting in the prediction of child conduct problems and callous–unemotional behaviors. *Dev. Psychopathol.* 28:757. 10.1017/S0954579416000298 27427804 PMC7557921

[B60] Mincy R. B. Jethwani M. Klempin S. (2015). *Failing Our Fathers : confronting the Crisis of Economically Vulnerable Nonresident Fathers.* Oxford: Oxford University Press, Incorporated.

[B61] Minuchin S. (1974). *Families and Family Therapy.* United States: Harvard University Press.

[B62] Muthén L. K. Muthén B. O. (2017). *Mplus User’s Guide.* 8th Edn. Los Angeles: Muthén & Muthén.

[B63] Nandy A. Nixon E. Quigley J. (2021). Observed and reported coparenting and toddlers’ adaptive functioning. *Infant Child Dev.* 30:e2226. 10.1002/icd.2226

[B64] Nepomnyaschy L. Miller D. P. Garasky S. Nanda N. (2014). Nonresident fathers and child food insecurity: Evidence from longitudinal data. *Soc. Serv. Rev.* 88 92–133. 10.1086/674970

[B65] Owen M. T. Caughy M. O. B. Hurst J. R. Amos M. Hasanizadeh N. Mata-Otero A.-M. (2013). Unique contributions of fathering to emerging self-regulation in low-income ethnic minority preschoolers. *Early Child Dev. Care* 183 464–482. 10.1080/03004430.2012.711594 23940412 PMC3739427

[B66] Pace G. T. Lee S. J. Grogan-Kaylor A. (2019). Spanking and young children’s socioemotional development in low-and middle-income countries. *Child Abuse Negl.* 88 84–95. 10.1016/j.chiabu.2018.11.003 30448642 PMC6357771

[B67] Paquette D. (2004). Theorizing the father-child relationship: Mechanisms and developmental outcomes. *Hum. Dev.* 47 193–219. 10.1159/000078723

[B68] Pilkauskas N. V. Schneider W. J. (2020). Father Involvement Among Nonresident Dads: Does Paternity Leave Matter? *J. Marr. Fam.* 82 1606–1624. 10.1111/jomf.12677

[B69] Pleck J. H. (2010). “Paternal involvement: Revised conceptualization and theoretical linkages with child outcomes.” in *The role of the father in child development*, 5th Edn, ed. Lamb M. E. (New York, NY: Wiley), 58–93.

[B70] Raffaelli M. Crockett L. J. Shen Y.-L. (2005). Developmental stability and change in self-regulation from childhood to adolescence. *J. Genet. Psychol.* 166 54–76. 10.3200/GNTP.166.1.54-76 15782678

[B71] Raver C. C. (2012). Low-income children’s self-regulation in the classroom: Scientific inquiry for social change. *Am. Psychol.* 67 681–689. 10.1037/a0030085 23163459 PMC4010145

[B72] Rochette É Bernier A. (2014). Parenting, family socioeconomic status, and child executive functioning: A longitudinal study. *Merrill Palmer Q.* 60 431–460. 10.13110/merrpalmquar1982.60.4.0431

[B73] Roggman L. A. Bradley R. H. Raikes H. H. (2013). “Fathers in family contexts” in *Handbook of Father Involvement: multidisciplinary Perspectives.* eds Cabrera N. Tamis-LeMonda C. (UK: Routledge). 186–201.

[B74] Roggman L. A. Fitzgerald H. E. Bradley R. H. Raikes H. (2012). *Methodological, Measurement, and Design Issues in Studying Fathers: an Interdisciplinary Perspective.* UK: Routledge. 17–46.

[B75] Roskam I. Stievenart M. Meunier J.-C. Noël M.-P. (2014). The development of children’s inhibition: Does parenting matter? *J. Exp. Child Psychol.* 122 166–182. 10.1016/j.jecp.2014.01.003 24607865

[B76] Roy K. Smith J. (2013). “Nonresident fathers, kin, and intergenerational parenting” in *Handbook of Father Involvement: multidisciplinary Perspectives.* eds Cabrera N. J. Tamis-LeMonda C. (UK: Routledge). 320–337.

[B77] Schoppe-Sullivan S. J. Fagan J. (2020). The evolution of fathering research in the 21st century: Persistent challenges, new directions. *J. Marr. Fam.* 82 175–197. 10.1111/jomf.12645

[B78] StGeorge J. Freeman E. (2017). Measurement of father–child rough-and-tumble play and its relations to child behavior. *Infant Ment. Health J.* 38 709–725. 10.1002/imhj.21676 29088498

[B79] Tamis-Lemonda C. S. McFadden K. E. (2010). “Fathers from low-income backgrounds: Myths and evidence” in *The Role of the Father in Child Development.* ed. Lamb M. E. (United States: John Wiley & Sons). 296–318.

[B80] Tanskanen A. O. Erola J. (2017). Do nonresident fathers compensate for a lack of household resources? The associations between paternal involvement and children’s cognitive and educational assessments in the UK. *Res. Soc. Stratif. Mobil.* 48 32–40. 10.1016/j.rssm.2017.01.002

[B81] Teubert D. Pinquart M. (2010). The association between coparenting and child adjustment: A meta-analysis. *Parent. Sci. Pract.* 10 286–307. 10.1080/15295192.2010.492040

[B82] Vogel C. A. Bradley R. H. Raikes H. H. Boller K. Shears J. K. (2006). Relation between father connectedness and child outcomes. *Parenting* 6 189–209. 10.1080/15295192.2006.9681305

[B83] Wåhlstedt C. Thorell L. B. Bohlin G. (2008). ADHD symptoms and executive function impairment: Early predictors of later behavioral problems. *Dev. Neuropsychol.* 33 160–178. 10.1080/87565640701884253 18443975

[B84] Winston P. Angel R. Burton L. Chase-Lansdale P. Cherlin A. Moffitt R. (1999). *Welfare, Children, and Families: A Three City Study, overview and Design.* Baltimore: Johns Hopkins University. https://web.jhu.edu/threecitystudy/images/overviewanddesign.pdf

[B85] Zelazo P. D. Carlson S. M. (2012). Hot and cool executive function in childhood and adolescence: Development and plasticity. *Child Dev. Perspect.* 6 354–360. 10.1111/j.1750-8606.2012.00246.x

[B86] Zvara B. J. Roger Mills-Koonce W. Cox M. Contributors F. L. P. K. (2016). Intimate partner violence, maternal gatekeeping, and child conduct problems. *Fam. Rel.* 65 647–660. 10.1111/fare.12221 28943690 PMC5603299

